# The acute effects of running on pelvic floor morphology and function in runners with and without running-induced stress urinary incontinence

**DOI:** 10.1007/s00192-023-05674-3

**Published:** 2023-11-22

**Authors:** Marie-Ève Bérubé, Linda McLean

**Affiliations:** School of Rehabilitation Sciences, University of Ottawa, 200 Lees Avenue E260C, Ottawa, ON K1N 6N5 Canada

**Keywords:** Dynamometry, Morphology, Pelvic floor, Running, Ultrasound, Urinary incontinence

## Abstract

**Introduction and hypothesis:**

The aim of this study was to examine the impact of a single running session on pelvic floor morphology and function in female runners, and to compare those with and without running-induced stress urinary incontinence (RI-SUI).

**Methods:**

This cross-sectional, observational study involved two groups: female runners who regularly experienced RI-SUI (*n* = 19) and runners who did not (*n* = 20). Pelvic floor muscle (PFM) properties were assessed using intravaginal dynamometry during maximal voluntary contractions (MVC) and during passive tissue elongation. The morphology of the pelvic floor was assessed at rest, during MVC and during maximal Valsalva maneuver (MVM) using 2D and 3D transperineal ultrasound imaging before and after a running protocol. Mixed-effects ANOVA models were used to compare all outcomes between groups and within-groups, including the interaction between group and time. Effect sizes were calculated.

**Results:**

No changes in PFM function assessed using intravaginal dynamometry were observed in either group after the run. Significant and large within-group differences were observed on ultrasound imaging. Specifically, the area and antero-posterior diameter of the levator hiatus were larger after the run, the bladder neck height was lower after the run, and the levator plate length was longer after the run (*p* ≤ 0.05). At the peak MVM and MVC, the bladder neck height was lower after the run than before the run (*p* ≤ 0.05). No between-group differences were observed for any outcomes.

**Conclusions:**

Running appears to cause transient strain of the passive tissues of the female pelvic floor in runners both with and without RI-SUI, whereas no concurrent changes are observed in PFM contractile function.

## Introduction

Stress urinary incontinence (SUI), the complaint of involuntary urine loss [1] that occurs when the bladder pressure exceeds the urethral capacity to remain closed, is the most commonly reported pelvic floor dysfunction during high-impact activities [2] and can limit females from participating in an active lifestyle [3, 4]. There is ongoing debate in the literature regarding the impact of exercise on pelvic floor function. Whereas some advocate that high-impact exercise is harmful and may induce strain on the structures that provide support [5], the evidence for harm is scant and some researchers encourage females to participate in high-intensity training, noting that risk is low and that exercise may improve pelvic floor support and strength [6]. Both hypotheses are supported by a review [2], which reported that females who engaged in physical activity had a 177% higher risk of reporting urinary incontinence than sedentary females, yet concurrently noted that exercise, when done at mild to moderate intensity, was protective of the pelvic floor, decreasing the odds and the risk of experiencing urinary incontinence [7].

As with SUI in the overall population, multiple factors, such as neuromuscular damage and structural damage to the urethra and the levator ani muscles, as well as their associated connective tissues [8, 9], are likely implicated in SUI experienced during athletic activities. Yet repetitive loading of the pelvic floor may, over a bout of exercise, lead to muscle fatigue and/or tissue strain, which additionally contributes to urine leakage during repetitive high-impact activities such as running [9]. Indeed, some researchers have reported that SUI during training occurs mainly during the middle or at the end of training sessions [10].

To our knowledge, no studies have investigated the acute, transient impact of running on pelvic organ support and pelvic floor muscle (PFM) function, or compared these effects between females who experience running-induced stress urinary incontinence (RI-SUI) and those who do not. Such robust biomechanical data are needed to fully understand the acute effects of running on pelvic floor morphology and function. Considering the absence of previous research in this area, the goal of this study was to provide critical insight into the impact of running exposure on pelvic floor morphology and function in female runners. We hypothesized that all runners would demonstrate evidence of levator ani muscle fatigue and a reduction in connective tissue support after the run, and that these changes would be more marked among runners with RI-SUI. The study was developed to generate new knowledge that would contribute to the development of targeted, evidence-based pelvic floor physiotherapy interventions for exercise-induced urine leakage, particularly during running.

## Materials and methods

### Design

This cross-sectional, observational study received prior approval from the local institutional research ethics board. Data were collected between June 2019 and March 2021, with a pause between March 2020 and January 2021 owing to laboratory closures caused by the COVID-19 pandemic.

### Participants

Female runners assigned female at birth, over 18 years of age, and with no known risk factors related to physical activity (as determined by the administration of the Physical Activity Readiness Questionnaire for Everyone (PAR-Q +) [11]) were recruited from the local community through local running groups and physiotherapy clinics. To meet the eligibility criteria, which were aimed at ensuring that runners had sufficient, regular, and prolonged exposure to high-impact loading of the pelvic floor through running, volunteers were required to run at least 5 km in under 50 min, twice a week, and to have maintained an average running distance over 10 km per week for a minimum of 1 year. Runners were recruited into two groups, intended to be matched by age and parity: runners who frequently experienced SUI (one or more episodes per week) while running and those who did not. Runners with SUI during running who reported using mitigation strategies (voiding their bladder before/during the run, running slower, avoiding hills, etc.) to avoid leakage were included if they leaked more than once per week when not using these strategies and still had a minimum one leakage episode while running per month while using these strategies. Participants were not excluded if they experienced occasional instances of urinary urgency during running without any associated leakage. Runners were excluded if they presented with a history of urogenital surgery or neurological disorders, if they reported symptoms of low energy deficiency as measured by the Low Energy Availability in Females Questionnaire (LEAF-Q) [12], if they were pregnant or if they had delivered a baby within the previous year, if they had a pelvic organ prolapse that was caudal to the hymen on a maximal Valsalva maneuver (MVM) in a standing position, or if they experienced pelvic pain to the extent that they were not able to undergo an intravaginal examination using a speculum. They were also excluded if they reported leakage associated with urgency during running. After providing written informed consent, all eligible participants provided demographic information including age, parity, obstetrical history (i.e., vaginal vs caesarean section, use of forceps or vacuum) and menopausal status, and were asked to complete the International Consultation on Incontinence—Female Lower Urinary Tract Symptoms Long Form module (ICIQ-FLUTS) [13], the Anal Incontinence Symptoms and Quality of Life module (ICIQ-B) [14], the Vaginal Symptoms module (ICIQ-VS) [15], and the International Physical Activity Questionnaire (IPAQ) [16].

### Laboratory assessment

The laboratory assessment was performed by a registered physiotherapist trained in pelvic health and with over 5 years of experience in this practice area, who also received over 50 h of training on intravaginal dynamometry and ultrasound imaging, and had over 2 years of experience using these instruments.

The morphology of the PFMs was assessed using 2D and 3D B-mode transperineal ultrasound imaging (USI) via a GE Voluson S6 system (General Electric, Toronto, Canada) with a standardized bladder volume between 100 and 200 mL. In a standardized standing position, static (3D) and dynamic (4D) volumes were acquired at rest and while the participant performed an MVM. 2D ultrasound videos were then recorded during MVM and maximal voluntary contraction (MVC) efforts of their PFMs. Next, still in standing, participants were instrumented with an intravaginal dynamometer and performed an MVC against arms opened to an anteroposterior diameter of 35 mm. In supine, passive forces were then recorded while the diameter of the dynamometer arms moved from 15 to 40 mm at a constant speed (50 mm/s). The elongation was held for 7 s before the arms returned to their initial position. Three repetitions of each task were performed.

Measures of PFM morphology in 3D included the area of the levator hiatus and its related antero-posterior (AP) and medio-lateral (ML) diameters both at rest and during peak MVM, as well as their change from the start to the peak position during the MVM. Signs of complete and partial levator avulsion were evaluated using the 3D rest volume in standing and the techniques described by Dietz et al. [17, 18]. The measures acquired in 2D included bladder neck (BN) height and levator plate (LP) length and their change from the start to the peak position during each task (MVC and MVM) [19, 20]. These measures have been found to have adequate reliability for use in research [21–23]. Measures of PFM function acquired through dynamometry included baseline force, relative peak force, rate of force development, and static endurance measured as the time from the initial force achieved during the MVC until force decreased by 35% [24, 25]. Passive tissue forces measured during tissue elongation included baseline force, relative peak resistance, rate of force development (stiffness), and the stress relaxation coefficient (SRc). The validity and reliability of these measures and all details of the standardized procedures can be found in previous publications [24, 25].

With a pre-weighed incontinence pad in situ and a Fitbit Charge 2 worn on the left wrist, the participant began a standardized running protocol on a treadmill (NordicTrack Commercial 2450). Participants increased the belt speed on the treadmill at a self-selected rate to move from walking to a slow jog at a speed of 7 km/h. At this speed, they ran for 2 min, then incremented the belt speed to 10 km/h for 2 min, and finally to 15 km/h for 2 min [26]. The participants were permitted to reduce the belt speed from 15 km/h if they did not feel safe or if they did not have the capacity to run at that speed. The participants were then asked to choose a self-selected speed at an intensity that they deemed to be hard (level 15–16 on the Borg Scale), and to run at that speed for 30 min. After the 30-min steady-state run, the participants ran for 30 s at 10 km/h and then at 7km/h. Participants were asked to report any instances of perceived leakage of urine while they ran. The ultrasound imaging and intravaginal dynamometry protocols were repeated as quickly as possible after the running protocol, with data collection completed within 15 min of the end of the run. The pad weight was recorded.

### Statistics

Using pilot data from the area of the levator hiatus at rest in standing recorded using transperineal USI before and after a 30-min running protocol from 8 continent runners (mean before 15.99 cm^2^, SD 2.32 cm^2^; mean after 17.63 cm^2^, SD 2.54 cm^2^), an a priori two-tailed power calculation using G*Power (version 3.0.10; α = 0.05; β = 0.80; effect size dz 0.67) estimated that a minimum of 20 continent runners would be necessary to detect within-group changes induced by the running protocol.

All demographic variables and outcomes were tested for normality using the Shapiro-Wilks test. Mixed-effects ANOVA models were used to compare all outcomes between groups (with or without RI-SUI) and within-groups (before and after the run) including the interaction between group and time. Residual plots were examined to ensure proper model fit, and effect sizes (Cohen’s d) were calculated using partial eta squared; d < 0.2 were considered small, 0.2 <  = d < 0.5 were considered moderate, and d >  = 0.8 were considered large [27]. Based on the observation that not all participants in the group with RI-SUI leaked urine during the protocol, secondary post-hoc exploratory analyses were performed using mixed-effects ANOVAs to evaluate effect sizes between runners with RI-SUI who leaked urine during the protocol vs runners with RI-SUI who did not leak during the protocol, and changes induced by the run within each group. Because of the exploratory nature of this research, the α level (α = 0.05) was not adjusted to account for multiple comparisons.

## Results

### Sample demographic information

**Fig. 1:**
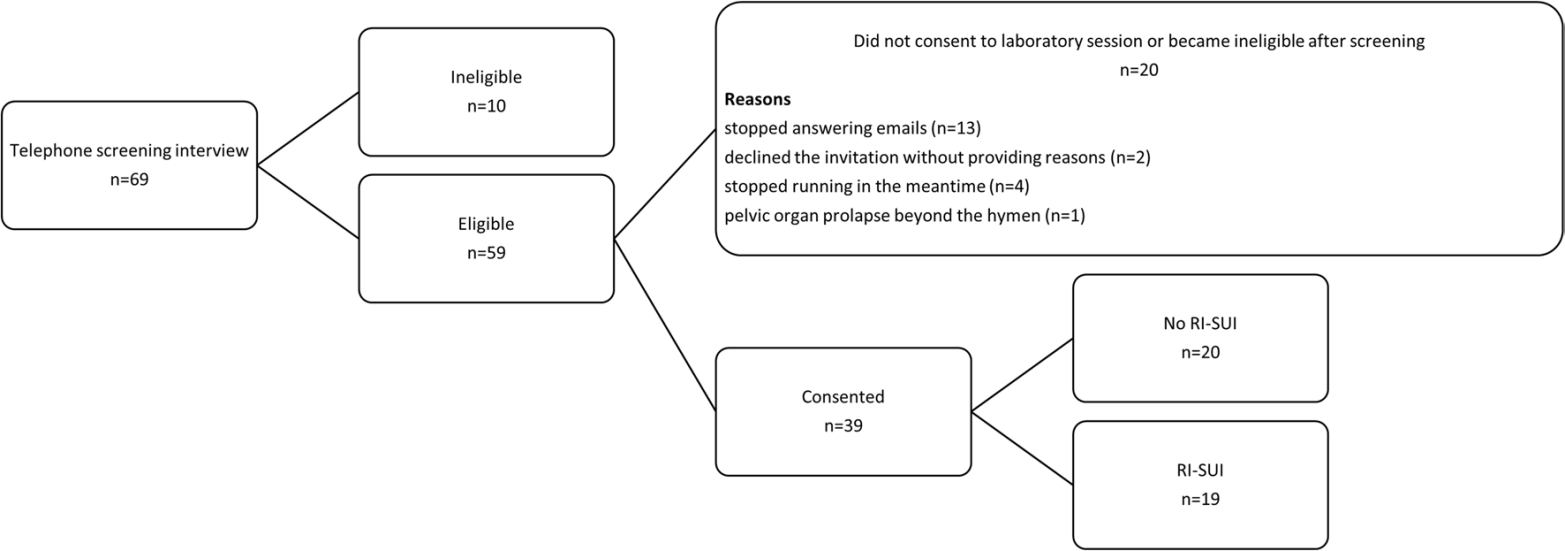
Flow diagram of the recruitment of the female runners with and without running-induced stress urinary incontinence (*RI-SUI*)

The recruitment flow diagram is presented in Fig. 1. Twenty participants reported being continent during running, reporting fewer than three lifetime episodes of urine leakage, and 19 runners reported experiencing frequent SUI during running. The groups were similar for most demographic outcomes (Table 1); however, the female runners without RI-SUI were slightly younger (*p* = 0.01) and ran slightly faster (*p* = 0.039) than the runners who reported RI-SUI, and although there was no difference in parity between the groups, there was a higher proportion of multiparous runners in the RI-SUI group (*p* = 0.013). The ICIQ-FLUTS UI subscale score was significantly higher (*p* ≤ 0.001) in runners with RI-SUI. The ICIQ-FLUTS scores indicated that 3 participants (1 without and 2 with RI-SUI) reported overactive bladder symptoms but none of them reported leakage associated with it during running. Although the runners with RI-SUI had greater pad weight gain after the running protocol than their continent counterparts (8 g versus 30.4 g), this was not statistically different between the groups. Seven out of the 19 runners with RI-SUI did not report being aware of any leakage during the running protocol and had a pad weight gain of less than 8 g over the course of the run. One continent participant had to discontinue the testing protocol after the running protocol as performing the MVM during the USI assessment caused a vasovagal response.

**Table 1:** Demographic information

	No SUI with running (*n* = 20)		SUI with running (*n* = 19)		Significance
	Mean/*n*	SD/%	Mean/*n*	SD/%	
Age (years)	36.2	7.9	43.9	10.6	0.01*
SUI with other activities (*n*, %)	8	40%	16	84%	**χ(1) = 8.046,** *p *= 0.005**
BMI (kg/m^2^)	22.0	2.3	24.2	5.0	0.184*
Waist to hip ratio	0.8	0.1	0.8	0.1	0.55*
Parous (n,%)	6	30%	11	58%	χ(1) = 3.083, *p* = 0.079**
Primipara (*n*, %)	3	15%	1	5%	χ(1) = 1.004, *p* = 0.316**
Multipara (*n*, %)	3	15%	10	53%	**χ(1) = 6.209, ***p* = 0.013**
Use of forceps (*n*, %)	1	5%	3	16%	χ(1) = 1.232, *p* = 0.267**
Use of suction (*n*, %)	0	0%	3	16%	χ(1) = 3.421, *p* = 0.064**
Signs of complete avulsion (*n*, %)	1	5%	0	0%	χ(1) = 0.975, *p* = 0.323**
Signs of partial avulsion (*n*, %)	3	15%	4	20%	χ(1) = 0.242, *p* = 0.622**
Menopause (*n*, %)	1	5%	3	16%	χ(1) = 1.232, *p* = 0.267**
Pelvic floor muscle training (*n*, %)	2	10%	6	32%	χ(1) = 2.783, *p* = 0.095**
ICIQ-FLUTS total score (/48)	5.0	4.2	9.9	3.3	< 0.001*
ICIQ-FLUTS F (/16)	2.7	2.2	3.3	2.1	0.351*
ICIQ-FLUTS V (/12)	0.7	2.0	0.8	1.3	0.322*
ICIQ-FLUTS I (/20)	1.7	2.0	5.8	2.3	< 0.001*
ICIQ-Bowel total score (1–75)	7.9	6.3	9.3	7.5	0.296*
ICIQ-Bowel pattern (1–21)	3.2	1.4	3.8	1.4	0.235*
ICIQ-Bowel control (/28)	2.2	2.7	2.8	3.3	0.365*
ICIQ-Bowel quality of life (/26)	2.5	4.6	2.7	3.6	0.513*
ICIQ-Vaginal symptoms (/53)	2.7	3.2	4.4	6.0	0.461*
ICIQ-Sexual Matters (/58)	6.9	9.8	3.8	7.9	0.478*
ICIQ-Vaginal quality of life (/10)	0.7	1.3	0.7	1.1	0.923*
IPAQ total activity (MET-min/week)	3,294.7	2,122.5	5,497.0	8,089.7	0.687*
IPAQ subtotal vigorous activities (MET-min/week)	1,278.0	918.2	1,708.9	3,099.0	0.531*
Self-selected running speed (km/h)	10.8	1.2	9.7	1.8	0.039***
Number of steps during running protocol	6,586.8	424.7	6,381.2	305.1	0.095***
Pad test (g)^a^	8.0	5.6	30.4	41.7	0.127*
Weekly running distance (km)	35.2	21.2	39.5	27.7	0.835*
Running experience (years)	15.1	9.2	15.9	12.0	0.792*

Values are presented as mean ± standard deviation (SD) or the frequency and percentage

*SUI* stress urinary incontinence, *BMI* body mass index, *ICIQ-FLUTS* International Consultation on Incontinence—Female Lower Urinary Tract Symptoms Long Form module, *IPAQ* International Physical Activity Questionnaire, *MET *metabolic equivalent of task

Significant differences (α =  ≤ 0.05) between groups are indicated in bold

Significance represents the level for between-group differences from the *Mann–Whitney *U* test, **the Chi-squared, or ***the two-sample *t* test

^a^One pad test was over 150 g and our scale had an upper range of 150 g; therefore, it was set to 150 g

### Ultrasound imaging outcomes

The morphological features of the levator hiatus at rest and during MVM are presented in Table 2. Significant and large main effects were found when comparing measures after the run with those measured before the run. At rest, the area (*p* = 0.002; d = 1.19) and AP diameter (*p* = 0.021; d = 0.84) of the levator hiatus were larger after the run for both groups but no change in levator hiatus morphology was observed during peak MVM when performed after the run compared with before the run. At rest, in both groups, the BN height was more caudal within the pelvis (*p* < 0.001; d = 2.09) and the LP was significantly longer (*p* < 0.001; d = 1.34) after the run than before the run. At peak MVC and peak MVM the BN was also more caudal after the run (*p* < 0.002; d = 1.20 and *p* < 0.001; d = 1.41 respectively) compared with before the run in both groups.

**Table 2:** Comparison of intravaginal dynamometry and ultrasound imaging outcomes before and after an acute bout of running between runners with and without running-induced stress urinary incontinence

	Before the run						After the run											
	No RI-SUI			RI-SUI			No RI-SUI			RI-SUI			Between-group		Within-group		Interaction	
	Mean	*n*	SD	Mean	*n*	SD	Mean	*n*	SD	Mean	*n*	SD	*p* value	Cohen's d	*p* value	Cohen's d	*p* value	Cohen's d
Dynamometry outcomes during MVC																		
Baseline (N)	7.90	17	2.68	7.62	12	2.79	7.28	17	2.59	7.27	12	2.16	0.875	0.06	0.172	0.54	0.702	0.14
Relative peak force (N)	3.23	17	1.56	4.21	12	1.29	3.73	17	1.89	4.16	12	1.36	0.232	0.47	0.158	0.56	0.096	0.66
Rate of force development (N/s)	8.20	17	7.09	11.21	12	3.89	8.86	17	7.88	10.29	12	4.84	0.357	0.36	0.792	0.11	0.125	0.61
Endurance (s)	2.23	16	0.55	2.58	12	0.82	2.12	16	1.19	2.33	12	0.70	0.336	0.39	0.288	0.42	0.670	0.17
Dynamometry outcomes during passive elongation																		
Baseline (N)	1.21	16	0.74	1.55	13	1.39	1.27	16	0.82	1.45	13	1.38	0.527	0.25	0.773	0.11	0.144	0.58
Relative peak force (N)	12.73	16	5.60	9.72	13	4.19	12.02	16	7.01	9.48	13	3.38	0.169	0.54	0.229	0.47	0.551	0.23
Stiffness (N/s)	34.25	16	10.96	25.50	13	11.09	31.58	16	11.60	25.14	13	8.60	0.056	0.77	0.228	0.47	0.355	0.36
Stress relaxation coefficient	-7.20	16	2.26	-7.54	13	2.59	-7.12	16	2.41	-6.87	13	2.30	0.947	0.00	0.548	0.24	0.638	0.18
3D ultrasound measurements at rest																		
Area (cm^2^)	15.65	18	4.29	18.00	17	5.35	16.37	18	4.88	18.84	17	5.16	0.153	0.51	**0.002**	1.19	0.792	0.09
AP diameter (cm)	5.23	18	0.80	5.52	17	0.77	5.36	18	0.86	5.68	17	0.74	0.257	0.40	**0.021**	0.84	0.727	0.13
ML diameter (cm)	3.86	18	0.46	4.23	17	0.73	3.86	18	0.45	4.21	17	0.64	0.068	0.66	0.870	0.06	0.883	0.06
3D ultrasound measurements on MVM																		
Area (cm^2^)	19.52	18	4.97	21.74	18	5.89	19.73	18	4.65	21.94	18	6.16	0.239	0.43	0.608	0.19	0.994	0.00
AP diameter (cm)	5.71	18	0.74	6.08	18	0.76	5.75	18	0.76	6.16	18	0.68	0.109	0.59	0.559	0.21	0.831	0.06
ML diameter (cm)	4.31	18	0.63	4.57	18	0.78	4.31	18	0.53	4.51	18	0.75	0.327	0.36	0.521	0.23	0.555	0.21
2D ultrasound measurements at rest																		
BN height (mm)	17.98	19	5.13	16.22	17	3.57	14.66	19	4.54	13.92	17	4.19	0.379	0.31	** < 0.001**	2.09	0.276	0.38
LP length (mm)	54.88	19	7.48	55.81	18	7.31	57.94	19	9.40	60.01	18	8.10	0.554	0.20	** < 0.001**	1.34	0.539	0.21
2D ultrasound measurements on MVC																		
BN height (mm)	20.83	18	4.88	19.57	17	3.91	18.45	18	5.01	18.65	17	4.27	0.721	0.13	**0.002**	1.20	0.135	0.53
LP length (mm)	45.37	18	8.05	45.52	18	6.23	45.60	18	8.75	45.02	18	5.91	0.929	0.00	0.768	0.11	0.443	0.26
2D ultrasound measurements on MVM																		
BN height (mm)	8.26	19	6.04	7.32	17	3.83	6.26	19	5.61	5.46	17	4.20	0.594	0.18	** < 0.001**	1.41	0.877	0.06
LP length (mm)	60.86	17	7.48	60.85	18	9.92	62.96	17	8.60	62.86	18	9.27	0.985	0.00	0.070	0.65	0.963	0.00

*RI-SUI* running-induced stress urinary incontinence, *MVC* maximal voluntary contraction, *MVM* maximal Valsalva maneuver, *2D* 2-dimensional, *3D* 3-dimensional, *BN* bladder neck, *LP* levator plate, *AP* antero-posterior, *ML* medio-lateral, *N/s* Newtons per second, *SD* standard deviation

Significant differences (α =  ≤ 0.05) are indicated in bold

The transient changes in morphological outcomes induced by MVC and MVM are presented in Table 3. There was more shortening of the LP during the MVC (*p* = 0.034; d = 0.76) and less lengthening of the LP during the MVM (*p* = 0.004; d = 1.08) after the run compared with before the run in both groups. There was also less caudal displacement of the BN during the MVM in both groups (*p* = 0.041; d = 0.73) after the run. No significant differences were observed between groups (RI-SUI vs control) and no interactions between group and time (before vs after the run) were observed for any morphological features measured from USI. Missing data (indicated in Tables 2 and 3) were due to poor image/video quality, which prevented the researcher from identifying landmarks (pubis symphysis, anorectal angle, anterior bladder neck) as indicated in the Tables.

**Table 3:** Assessment of the change in position of the bladder neck and levator plate on ultrasound imaging between runners with and those without running-induced stress urinary incontinence

	Before the run						After the run											
	No RI-SUI			RI-SUI			No RI-SUI			RI-SUI			Between-group		Within-group		Interaction	
	Mean	*n*	SD	Mean	*n*	SD	Mean	*n*	SD	Mean	*n*	SD	*p* value	Cohen's d	*p* value	Cohen's d	*p* value	Cohen's d
3D ultrasound change on MVM																		
Area (cm^2^)	3.23	18	2.16	2.92	18	2.20	3.01	18	2.01	2.09	18	2.34	0.371	0.33	0.127	0.57	0.379	0.32
AP diameter (cm)	0.46	18	0.62	0.42	18	0.54	0.35	18	0.55	0.27	18	0.36	0.694	0.14	0.136	0.55	0.809	0.09
ML diameter (cm)	0.33	18	0.28	0.30	18	0.23	0.32	18	0.23	0.26	18	0.33	0.574	0.20	0.527	0.23	0.685	0.14
2D ultrasound change on MVC																		
BN height (mm)	2.41	18	2.75	4.36	17	4.10	3.57	18	2.94	4.48	17	2.97	0.163	0.50	0.143	0.52	0.234	0.42
LP length (mm)	-9.70	18	4.60	-11.06	18	4.71	-10.84	18	5.65	-12.95	18	5.48	0.276	0.38	**0.034**	0.76	0.583	0.19
2D ultrasound change on MVM																		
BN height (mm)	-9.71	19	4.00	-8.90	17	4.33	-8.40	19	3.73	-8.46	17	4.04	0.77	0.11	**0.041**	0.73	0.296	0.36
LP length (mm)	6.00	17	7.34	5.05	18	5.08	4.63	17	5.33	2.84	18	3.77	0.444	0.27	**0.004**	1.08	0.471	0.26

Negative values indicate a shortening of the levator plate or a caudal descent of BN height

*RI-SUI* running-induced stress urinary incontinence, *MVC* maximal voluntary contraction, *MVM* maximal Valsalva maneuver, *2D* 2-dimensional, *3D* 3-dimensional, *BN* bladder neck, *LP* levator plate, *AP* antero-posterior, *ML* medio-lateral, *SD* standard deviation

Significant differences (α =  ≤ 0.05) are indicated in bold

### Intravaginal dynamometry outcomes

The dynamometry outcomes obtained from the MVC and passive tissue elongation before and after the run are presented in Table 2. No significant between- or within-group differences were found; however, moderate effect sizes were observed for the interaction between group (RI-SUI vs controls) and time (before vs after the run) for strength (relative peak force; d = 0.66) and power (rate of force development; d = 0.61) achieved during peak MVC. Although the strength and power achieved during the MVC tended to increase after the run among runners without RI-SUI, relative to their baseline, power tended to decrease, and strength remained unchanged among runners with RI-SUI after the run. During passive tissue elongation, the stiffness and relative peak force tended to be lower in runners with RI-SUI compared with their continent counterparts (*p* = 0.056; d = 0.77 and *p* = 0.169; d = 0.54 respectively), and tended to decrease after the run for both groups (d = 0.47 and d = 0.47 respectively). Missing data as reported in Table 2 were due to technical difficulties with the hardware of the intravaginal dynamometer, which prevented the researcher from collecting a complete data set in some cases.

### Post-hoc comparison of runners with RI-SUI whose leakage was provoked vs those whose leakage was not provoked during the running protocol

As presented in Table 4, there was a large between-group (those with RI-SUI who leaked vs those with RI-SUI who did not leak) and time (before vs after the run) interaction effect in terms of the peak position of the BN during the MVC (*p* = 0.045; d = 0.92). There was also a large between-group effect for LP length at rest and LP length during peak MVM (*p* = 0.013; d = 1.40 and *p* = 0.039; d = 1.12 respectively). Indeed, among those with RI-SUI whose leakage was provoked during the running protocol, the BN position sat more caudal within the pelvis, particularly after the run, and the LP was more elongated. There were also large effect sizes when relative peak force (*p* = 0.210; d = 0.80) and stiffness (*p* = 0.224; d = 0.78) recorded during passive tissue elongation were compared between the groups—the runners with RI-SUI whose leakage was provoked during the running protocol tended to demonstrate lower resistance of their paravaginal tissues to passive stretch imposed through intravaginal dynamometry.

**Table 4:** The effects of running on intravaginal dynamometry and ultrasound imaging outcomes in runners with running-induced stress urinary incontinence, stratified by whether or not leakage was provoked by the running protocol

	Before the run						After the run											
	Did not leak			Leaked			Did not leak			Leaked			Between-group		Within-group		Interaction	
	Mean	*n*	SD	Mean	*n*	SD	Mean	*n*	SD	Mean	*n*	SD	*p* value	Cohen's d	*p* value	Cohen's d	*p* value	Cohen's d
Dynamometry outcomes during MVC																		
Relative peak force (N)	4.38	5	1.56	4.08	7	1.19	4.37	5	1.50	4.02	7	1.36	0.689	0.26	0.860	0.11	0.902	0.09
Rate of force development (N/s)	11.66	5	3.18	10.89	7	4.55	11.35	5	4.23	9.53	7	5.42	0.624	0.32	0.254	0.77	0.463	0.48
Dynamometry outcomes during passive elongation																		
Relative peak force (N)	11.49	5	6.04	8.61	8	2.36	11.07	5	4.95	8.48	8	1.62	0.210	0.80	0.587	0.34	0.770	0.18
Stiffness (N/s)	29.48	5	14.37	23.02	8	8.63	29.27	5	11.51	22.56	8	5.58	0.224	0.78	0.884	0.09	0.957	0.00
3D ultrasound measurements at rest																		
Area (cm^2^)	17.04	6	6.53	18.53	11	4.85	17.48	6	5.55	19.59	11	5.05	0.513	0.35	0.066	1.02	0.433	0.41
3D ultrasound measurements on MVM																		
Area (cm^2^)	21.62	5	7.56	21.80	11	5.40	21.64	5	8.57	22.08	11	5.24	0.926	0.06	0.831	0.11	0.851	0.11
2D ultrasound measurements at rest																		
BN height (mm)	15.80	6	2.27	16.44	11	4.20	14.94	6	3.30	13.37	11	4.66	0.812	0.13	**0.006**	1.64	0.094	0.92
LP length (mm)	51.18	6	6.88	58.12	12	6.60	53.48	6	5.63	63.28	12	7.21	**0.013**	1.40	**0.026**	1.22	0.363	0.47
2D ultrasound measurements on MVC																		
BN height (mm)	18.14	7	3.88	20.58	10	3.79	18.76	7	3.06	18.58	10	5.11	0.572	0.30	0.268	0.59	**0.045**	1.13
LP length (mm)	42.90	7	6.81	47.19	11	5.51	41.59	7	5.62	47.21	11	5.18	0.085	0.92	0.283	0.56	0.270	0.57
2D ultrasound measurements on MVM																		
BN height (mm)	8.89	6	2.68	6.46	11	4.19	7.71	6	2.48	4.23	11	4.52	0.141	0.80	**0.003**	1.84	0.290	0.57
LP length (mm)	56.23	6	11.25	63.17	12	8.78	55.55	6	7.42	66.51	12	8.00	**0.039**	1.12	0.478	0.36	0.288	0.55

*MVC* maximal voluntary contraction, *MVM* maximal Valsalva maneuver, *2D* 2-dimensional, *3D* 3-dimensional, *BN* bladder neck, *LP* levator plate, *N/s,* Newtons per second, *SD* standard deviation

Significant differences (α =  ≤ 0.05) are indicated in bold

## Discussion

Both runners with and without RI-SUI experienced significant transient changes, particularly in the morphology of their pelvic floor as measured by USI after they completed a 37-min treadmill running protocol. The changes induced by running appear to predominantly affect the passive support system, as evidenced by the larger area and AP diameter of the levator hiatus, the more caudal position of the BN, and the elongated LP observed in quiet standing after running compared with baseline. Although PFM function, as measured by intravaginal dynamometry, was not significantly affected by the run, the directionality of the observed effects was consistent with the morphological changes. Specifically, the relative peak force and stiffness measured during passive tissue elongation tended to be lower after the run than before the run. And, although differences were not statistically significant, the runners who experienced RI-SUI tended to generate less power after running than before the run, whereas their strength (relative peak force) did not change. Conversely, the runners without RI-SUI tended to generate greater force and more power after the run than before the run. These findings suggest that an acute bout of running might result in strain of the passive tissues within the pelvis, which was observed in all female runners, regardless of whether they experience urine leakage during running. Such changes are likely related to transient changes in the viscoelastic characteristics of the tissues after an episode of repetitive loading—the tissues likely return to their prestressed state assuming that there is no underlying damage; however, the cumulative effects of repeated running bouts should be investigated.

### Ultrasound imaging

Running caused significant transient morphological changes in pelvic morphology, as measured by 2D and 3D USI, mainly reflecting elongation of the passive supporting structures. Indeed, the passive support structures appeared to be equally affected by the activity in both runners with and those without RI-SUI. Based on these findings, it is plausible that the initial morphological features of the levator hiatus, BN, and LP may serve as a predisposing factor for the development of RI-SUI over the course of a run. It is possible that some threshold exists for the levator hiatus area, LP length, BN height, or a combination of these factors beyond which the passive support to the urethra is no longer sufficient to support and compress it between the anterior vaginal wall and the pubic symphysis to prevent urine leakage. Consistent with this theory, a recent study investigating the likelihood of SUI symptoms being cured by a PFM training intervention found that a resting BN position lower than 14.3 mm in standing, as measured on 2D USI, was predictive of treatment failure [28]. In the current study, continent runners had a resting BN height that was above this threshold both before and after the run (17.98 mm before vs 14.66 mm after); however, the BN of runners with RI-SUI dropped below this threshold after the run (16.22 mm before vs 13.92 mm after). Although the BN may descend over the course of a run in all female runners, leakage may occur when the BN position ultimately reaches some failure point [28]. This theory is consistent with findings that athletes most often leak urine toward the middle and end of a training session [10].

The significant increase in the extent of shortening of the LP observed during the MVC after the run, and the significant decrease in the extent of lengthening of the LP during the MVM after the run are likely due to a transient increase in the length of the LP. With a longer LP length at rest, the LP had to shorten more during the MVC to achieve the same end-MVC length observed before the run, and similarly the LP did not have as much room to lengthen during MVM before it reached its ultimate endpoint. As with the LP, the significant reduction in both groups in the extent of BN descent observed during the MVM after the run can be attributed to the lower resting position of the BN after the run, which was already close to its maximal descent.

### Intravaginal dynamometry

Although not significant, the trends observed on dynamometry are worth discussing, as the effect sizes were moderate to large; yet these results should be interpreted with caution. Runners with RI-SUI demonstrated a 9% reduction in the contractile power of their levator ani muscles after running, whereas their strength remained unchanged. In contrast, the runners without RI-SUI tended to have greater strength (14% increase) and power (8% increase) after the run than before the run. These results align somewhat with previous findings among nulliparous females with mild SUI, where a mean reduction in maximal vaginal closure pressure induced by MVC of the levator ani muscles was substantive (-20%) but not significant following a 90-min exercise session that included 20 min of running [29]. Also, in line with the current findings, there were changes neither in vaginal resting pressure nor in the time over which an MVC could be sustained (i.e., static endurance) following the exercise session [29]. The current results are also consistent with findings that, following a 20-min as many repetitions as possible (AMRAP) workout, strenuous exercisers (27.7% with symptoms of urine leakage) demonstrated significantly more vaginal descent and significantly lower vaginal resting pressure [30], without concurrent changes in maximal vaginal closure pressure during MVC after the exercise [30]. A control group of nonstrenuous exercisers (8.57% with symptoms of urine leakage) completed a 20-min walk, and no differences were observed between the strenuous exercisers and the control group in terms of effect on pelvic organ support and vaginal closure pressure despite the different intensity of the exercise [30]. Both the current study and these previous results reinforce the idea that an acute bout of exercise may have a greater impact on passive pelvic organ support rather than PFM contractile function.

Although not significant, stiffness and peak passive force measured through dynamometry tended to be compromised in the runners with RI-SUI relative to their continent counterparts, which align with the observed morphological changes in all runners. Although both groups demonstrated similar reductions in stiffness and peak passive forces after completing the running protocol, the group with RI-SUI had lower absolute stiffness and lower peak passive forces after the running protocol. Further, the runners with RI-SUI whose leakage was provoked during the running protocol appeared to experience greater strain than those with RI-SUI whose leakage was not provoked. More research with a larger sample size is needed to establish the contribution of the loss of passive tissue stiffness in RI-SUI.

### Limitations

This study focused on experienced female runners and thus the findings may not be generalizable to infrequent runners. The sample included middle-aged nulliparous and parous, mostly white, healthy females. Although the outcomes of this investigation might be transferable to other athletes who engage in running as a component of other physical activities, the results may not be applicable to all athletes. Whereas the runners without RI-SUI selected a faster treadmill speed than their incontinent counterparts (10.8 km/h versus 9.7 km/h), step counts during the running protocol were not different between the groups, suggesting that, even though loading may have been higher in the continent group, the pelvic tissues were exposed to a similar number of loading cycles in both groups with comparable levels of exertion (15–16 on the Borg Scale). Indeed, had the runners with RI-SUI run at the same intensity as their continent counterparts, the observed changes may have been greater.

Vaginal birth is a known risk factor for SUI, and it impacts the morphology and functioning of the PFMs. Although efforts were made to balance parity as a dichotomous variable between the recruited groups, there was a higher proportion of female runners with multiple childbirths in the RI-SUI category in contrast to those without. Nonetheless, an equal number of participants in both groups (*n* = 4) exhibited levator avulsion (partial or complete). Additional alterations in the pelvic floor linked to parity such as nerve damage might have played a role in affecting the findings of this investigation.

Neural, metabolic, and vascular components of the continence system could impact differences in the force generation capacity of the PFMs after the run; however, these were not assessed in the present study. Indeed, although PFM surface electromyography (EMG) was pilot tested as an outcome to be measured concurrently with intravaginal dynamometry in this study, it was deemed not to be feasible, as the arms of the dynamometer caused significant motion artifact in the EMG signals, intravaginal electrodes were dislodged by the dynamometer arms, and adhesive electrodes lifted, particularly after the run, owing to perspiration, urine, and/or vaginal secretions.

Not all runners with RI-SUI leaked urine during the protocol. A longer or more strenuous protocol may have induced greater changes in morphology and function than was observed here. Although the sample size was small, this hypothesis is supported by the exploratory post-hoc analysis presented in Table 4. Among the runners with RI-SUI, those whose leakage was provoked during the running protocol, findings point to greater reductions in pelvic organ support compared with those with RI-SUI who did not experience leakage during the protocol. Although participants were evaluated quickly after the running protocol (within 15 min), it is possible that the passive PFM tissue properties may have recovered somewhat by the time the passive tissue elongation was performed. As such, the extent of tissue strain induced by the running protocol may be larger than that reported here.

A final limitation of this study is the extent of missing data that resulted from hardware failure in the intravaginal dynamometer and poor image quality during USI, which resulted in a smaller sample size for some outcomes. The interpretation of the results is also subject to the limitations of the instruments themselves. For instance, although intravaginal dynamometry is considered a gold standard for the measurement of PFM active and passive tissue properties, it remains a coarse measurement as it is influenced by all closure forces acting on the dynamometer arms [31, 32]. USI measurements can be influenced by changes in the angle of the transducer during acquisition and the incorrect identification of landmarks [31].

That said, the innovative approach used in this study to investigate the effects of an acute bout of running on active and passive pelvic floor support structures contributes to our understanding of RI-SUI. The findings point to practical approaches that focus on enhancing pelvic floor support such as PFM training and the use of pessaries to effectively mitigate or prevent symptoms of athletic incontinence in females. If pelvic floor support is enhanced prior to an acute bout of running, it might prevent the pelvic structures from descending below a critical threshold that leads to the failure of the continence system over the course of a run.

## Conclusion

The findings of this study indicate that a 37-min bout of treadmill running causes transient strain in the passive tissues in the pelvic floor, leading to significant loss of pelvic organ support among runners with and without RI-SUI. The findings revealed no evidence to support the hypothesis that the running protocol induced contractile fatigue in the PFMs. Future research should be aimed at investigating the time course of morphological changes that occur during running, as well as recovery profiles.

## Data Availability

All data relevant to the study are included in the article. Should any raw data files be needed in another format they are available from the corresponding author upon reasonable request.

## Abbreviations

AP: Antero-posterior
BN: Bladder neck
LH: Levator hiatus
LP: Levator plate
ML: Medio-lateral
MVM: Maximal Valsalva maneuver
MVC: Maximal voluntary contraction
PFM: Pelvic floor muscle
RI-SUI: Running-induced stress urinary incontinence
SUI: Stress urinary incontinence
USI: Ultrasound imaging
